# Spirit of the British and American Periodicals, &c.

**Published:** 1844-01-01

**Authors:** 


### Spirit of the British and American Periodicals, &c.

### SURGERY.

#### Removal of the Uterus by the Large Abdominal Section.

We would not willingly be accused of repressing attempts to enlarge the domain of successful operative surgery. But it is well to check the passion which is prone to prevail epidemically for great and hazardous operations. The removal of diseased ovaria is one of this description. Were we to trust to some recent Reports this would seem to be as easy as amputation of a finger and rather more successful. But, if the proceeding is resorted to as freely as it seems likely to be, we venture to predict that a liberal discount of disasters must be deducted from the sum-total of success. The following case is in point.

*Case.*—J. B. æt. 46, unmarried, under Mr. Heath's care, in the Manchester Union Hospital.

For four years she had suffered from periodical discharges of blood per vaginam. Twelve months ago, she perceived a tumor in the left hypochondriac region, about the size of a large orange, which rapidly increased in size.

The abdomen resembled that of a woman seven months gone in pregnancy—tumor extending, in the median line, from the pelvis to a little above the umbilicus—firm—and moveable. Os tincæ situated somewhat forward and closed, fissure transverse, cervix pyriform. Some motion transmitted from tumor when grasped to cervix.

It was the unanimous opinion of the consultants that the tumor was ovarian, and fairly adapted for extirpation by the large abdominal section. After preparatory treatment, that section was made, and then—

"The tumor now came into view, and was recognized as the uterus distended by solid matter; and this was rendered more certain by the introduction of a trocar. The size and solidity, with the rapid growth of the tumor, and the probable effects which would be produced by the next periodical discharge of blood, determined me at once to effect its removal *en masse*. Having passed my hand over the fundus of the uterus, and behind it, I raised it from the abdominal cavity, when it was sustained by Mr. Goodlad, while two double ligatures were passed, by means of a sharp-pointed aneurism-needle, through the cervix uteri, immediately below the circumference of the tumor. Each ligature was then firmly tied, so as to include one half of the neck of the womb and broad ligaments. The parts were then excised and removed. No bleeding ensued from the cut surface; indeed, throughout the operation, not more than three ounces of blood were lost; and after the first division of the skin, few complaints of suffering were made by the patient herself.

"The intestines, which had escaped, were replaced *in situ*, and the abdominal parietes brought together by the interrupted suture at seven points."

The patient lived 17 hours, sinking rather than presenting acute symptoms.

*Dissection.*—There was partial adhesion of the wound. Spots of increased vascularity, and distention of the bowels. Fourteen ounces of blood in the cavity of the abdomen, but the source of the hæmorrhage not obvious—the ligatures still lightly constricting the remains of the uterus, which comprised about two inches of it.

The uterus, with the tumor, weighed six pounds—it was smooth and globular—the walls of the organ three-quarters of an inch in thickness—the tumor itself fibrous, and growing from the muscular structure immediately beneath the mucous membrane at the fundus.

Now, without going into the merits of the case, it is enough for our purpose to observe, that the medical officers of a public charity agree unanimously to remove as ovarian tumor, an uterus with a fibrous growth in it—evidence enough of the uncertainty which too often attends the diagnosis of these cases.—*Medical Gazette*, Dec. 8, 1843.

---

### REMOVAL OF A DROPSICAL OVARIUM, ENTIRE, BY THE LARGE ABDOMINAL SECTION. BY D. H. WALNE, SURGEON.

On the 27th June, 1843, a Miss A. K. from the country, in the 20th year of her age, consulted Mr. Walne for a dropsical disease of one of the ovaries. Mr. W. found the abdomen as prominent as at the full period of pregnancy, with greater fulness towards the loins. The tumefaction was circumscribed, and distinctly fluctuating; health in all respects good—no sign of general dropsy—uterus, and its functions, perfectly natural—had never been tapped—general figure slender—some emaciation—still she measured 40 inches in circumference. Mr. W. proposed excision of the diseased ovary, as the only alternative. After considerable delay it was finally arranged that the operation should take place on the 12th of Sept. The usual preparatory measures were duly attended to.

Sept. 12. At 3 o'clock P.M. a copious enema of warm water ensured the clearance of the bowels. Drs. Blundell and H. Davies, Messrs. Vincent, Beale, Burrows, Camplin, Hitchman and Law, having assembled, and all the necessary preparations being complete, about a quarter before five o'clock, Miss K. seated herself firmly at the end of a couch. The pillows and bandage being duly adjusted, Mr. Beale took his post on the right, Mr. Law on the left of the patient, the former to manage the integuments, the latter the tumor. The operator sat obliquely facing the end of the couch. Mr. Walne made the exploratory small opening of an inch and a half in the linea alba and below the umbilicus—the abdominal cavity was soon entered, and the state of the cyst was ascertained by the finger. He then divided the integuments from above downwards in the median line, slightly deviating to the left at the umbilicus, and having reached the preliminary section at its upper end, he then prolonged the division downwards to the requisite extent—the incision was 14 inches in length. Then, with the curved probe-pointed bistoury, he divided the peritoneum from within to a like extent. An enormous cyst gradually advanced through the wound. The broad uterine ligament of the left side constituted its pedicle, through which the operator thrust from behind a needle armed with strong silk twist. The two halves of the pedicle were separately and tightly tied, and then it was divided between the ligatures and the tumor—the latter, weighing 28 lbs., being removed without impediment from adhesion. No bleeding ensued from the division of the pedicle which was firmly tied—13 interrupted sutures were applied—then pads of lint on each side of, but a little away from, the wound, and over these, strips of plaister, all being secured by a bandage. The case went on rapidly progressing towards perfect health with scarcely one single unfavourable symptom. On the 29th (17 days after the operation) the patient walked across the room, leaning on the nurse's arm, and the next day did so repeatedly without support. *Dimensions of tumor.*—When lying, its circumference measured horizontally 3 ft., 8½ inches vertically, lengthwise 3 ft. 2 in. Vertically across 2 ft. 10½ inches—28 lbs. imperial weight.

---

### CASE OF OVARIAN TUMORS—BOTH REMOVED AT THE SAME OPERATION. By JOHN L. ATLEE, M.D., Lancaster.

Early in the month of June, 1843, a lady under 30 years of age, who had never been married, became the subject of this operation. She had suffered from ascites for the last three years, for which she had been tapped four times, the ovarian tumors having remained undetected till after the third tapping. Dr. A., convinced that these large ovaria were the cause of the dropsy, proposed to remove them—the patient consented.

An incision was made into the abdominal cavity, about nine inches in length, in the course of the linea alba, and commencing at the pubes. The left ovarian tumor was found attached merely by the broad ligament, and floated free in the abdomen; while the tumor on the right side, adhering about two-thirds of its extent to the brim of the pelvis and the omentum, required some careful dissection in its removal. They were both removed without much hæmorrhage; and this large wound, which was brought together by the interrupted suture, is now, seven weeks after the operation, completely united, with the exception of the lower end, where the ligatures upon the ligament still remain. The patient recovered perfectly without an unfavorable symptom.—*New York Journal of Medicine,* Sept. 1843.

---

Phlebitis, with Remarks on its Pathology and Treatment. By N. Chapman, M.D., Professor of Medicine in the University of Pennsylvania.

When phlebitis occurs in an external or superficial vessel, there is pain, much increased on pressure, with swelling, stiffness, and a streak of redness along its course—the affection almost always proceeding towards the heart—there is also constitutional disturbance—the pain becomes more poignant, and the tumefaction enormous—if it occur in a limb, the latter becomes of more than double its natural size, skin tight, smooth, even glossy, and rather white than red, or purplish. The fever now becomes typhoid, marked by great prostration, pulse sometimes intermittent, or otherwise very irregular, nausea and vomitings, præcordial uneasiness, hurried respiration, deep sighing and dejection of spirits, foul coated tongue, &c. Chills or rigors, or rheumatic-like aches in the joints, or wandering about the cavities of the body. According to Dr. Chapman, spontaneous phlebitis very rarely occurs—he never saw a single case of it. It generally proceeds from some mechanical injury done to the vein by venæsection, amputation, the extirpation of tumors, &c. The tying of varicose veins has sometimes occasioned it. Compression from adjacent tumors also induces it—much is also imputed to the introduction of a virus into the circulation by inoculation or otherwise. As a common cause is also held the absorption of pus or acrid fluid from foul ulcers, or the secretions of various surfaces. The Doctor states that, phlebitis is owing almost exclusively to a wound of the vessel, and that it is perhaps never directly occasioned by a virus. There are times, and individuals, at all times, when the slightest incision or puncture, or even a scratch, will lead to the most disastrous results of this nature from the cleanest instruments. Every one knows that, at particular seasons, phlebitis is very apt to follow venæsection. The instances of tumefaction from the insertion of a virus he considers to be affections of the lymphatics, utterly denying to veins any absorbing power whatever (!!) The prognosis in phlebitis is in general very unfavorable. According to Hunter, in dissections of cases which have died from phlebitis, suppuration and ulceration have been found. Beginning usually in the lining tissue, the inflammation may pervade the whole of the vascular parietes. We also have extravasation of lymph, either on the surfaces, or, as the author says, "intertissual." Collections of pus and of lymph are sometimes detected in the calibre of the vessel to such an amount as to obstruct the circulation. Adhesions of the sides of the vessel have sometimes produced a total obliteration. For the extensive constitutional disturbance, almost always evincing a tendency to typhoid debility and vitiation, which sometimes occurs in phlebitis, the Doctor is totally unable to account satisfactorily. If it could be proved, he says, that the local inflammation was widely diffused through the veins, so as to embrace a large portion of them, then there might be some means of accounting for the enormous mischief produced. Such, however, is not the case, the inflammation being for the most part restricted to a few inches from the wound. The phenomenon he conceives is only to be accounted for on the supposition of the "sympathies of the rest of the veins with the affected one." What a convenient sort of thing this thing of sympathy has proved in the hands of the nonplussed medical philosopher! If we recollect rightly, Dr. Chapman it is, who, in his accounting for the modus operandi of medicinal substances,* invokes the aid and the exclusive aid of his favorite *sympathy*. The comparatively gross idea of medicines producing their sanatory effects by their being absorbed into the system he utterly repudiates. The mode of treating phlebitis proposed by Dr. Chapman, not differing in any particular from that usually employed, we shall here close our notice of this article.—*Abstracted from the American Journal of the Medical Sciences*, July, 1843.

---

### A Case of Spina Bifida successfully treated by repeated Punctures; with Practical Remarks. By Alexander H. Stevens, M.D.

Oct. 17, 1837. An infant, eight months old, was operated on by Dr. Stevens this morning for spina bifida. The tumor was seated over the upper part of sacrum, about 3½ inches broad from side to side, the same nearly in a vertical direction, and rising about two inches above the surrounding surface. The covering of the tumor was not healthy skin, but a peculiar thin membrane of a whitish colour, traversed by numerous vessels, like varicose capillary veins. The whole swelling was somewhat pendulous, narrower at its base. It had been once punctured with a needle, and the wound is now covered with a scab. In the first operation it was punctured with an iris knife, and about four ounces of clear serum, with a few drops of blood, were discharged. The child did not appear to suffer—a slight sinking was produced at the anterior fontanelle. On the 20th and 21st it was again punctured, and about five ounces of serum were discharged. The tumor now appeared shrivelled. Oct. 30th. The fluid continued to ooze for nearly 24 hours after the last punctures. Recently the sac of the tumor has become inflamed; and within two days the child has kept her left leg drawn up, and cried when disturbed. Tumor is kept wet with a spirit lotion—anterior fontanelle much depressed. No further puncturing. On the following Summer the sac of the water had disappeared, and all that remained was a small bunch of indurated integuments.

*Remarks*.—The mode of obviating syncope and spasms in this operation is by drawing off the water slowly, leaving some in the sac, keeping patient in the horizontal position, and making pressure on tumor and head.—*New York Journal of Medicine, Sept.* 1843.

---

### On Electro-Puncture in Hydrocele. By F. C. Stewart, M.D.

The idea of this mode of treatment originated with Dr. Pecchioli, an Italian

---

* See his work on Therapeutics.

surgeon, attached to the hospital of Sienna. The subject of his first case was a young man of a good constitution, who had had a simple double hydrocele for three years, without any apparent cause. Dr. P. introduced four needles into the scrotum, two on each side, one at the summit, and one at the base of each tumor; this enabled him to pass a galvanic current through the sacs, as soon as the needles were brought into contact with the respective poles of a small battery. The application was continued for five minutes, the patient feeling acute pain in the right testicle. On withdrawing the needles the tumors were much diminished, and after five hours they were almost empty. Towards evening, however, there was great heat and redness of the part, and the fluid was re-accumulated to its former amount. The operation was repeated after some days, and with a similar result; but, after a third operation, the fluid became permanently absorbed. Dr. Leroy, a Parisian surgeon, repeated a similar experiment at the Hôtel Dieu, on a man 70 years old, with a hydrocele on the right side. He caused a small battery, of 16 pairs of plates, two inches square, to act on the hydrocele, through two needles, placed, one in the subcutaneous cellular tissue of the scrotum, and the other penetrating into the cavity of the tunica vaginalis. In two days after, the tumor had entirely disappeared. A re-appearance of a little fluid rendered the repetition of the operation necessary twice afterwards, once for twenty minutes, and the other for ten. There was some pain. Dr. Stewart repeated the experiment on another patient, and, he thinks, with success. He gives the following directions. The needles should be at least four inches in length—and fine. After greasing—introduce cautiously, one into the subcutaneous cellular tissue of scrotum, at the summit of tumor, the other into the cavity of the tun. vag. near its base. The battery, which may be composed of from twelve to twenty pairs of plates of between two and three inches diameter, will now be brought into action by the addition of the acid solution (℥ij. of sulphuric acid to a quart of water to begin with), and the poles will be placed in contact with, and kept permanently applied to, the eyes of the needles, already introduced into the scrotum. According to Dr. S., the operation should be continued for forty minutes or more, and a regular supply of electricity kept up by the occasional addition of a few drops of acid to the solution in the trough. The patient should maintain the recumbent position for several days.—*New York Journal of Medicine*, Jan. 1843.

---

### Warm Injections in Stricture.

Mr. Hudson, on this occasion, uses a catheter with its orifice at the extremity, not at the side, and for convenience a stop-cocked syringe, holding about an ounce, is fitted to its other extremity. He fills the syringe with some warm bland liquid (oil or barley water), and then connects it with the catheter, and gently passes the latter down to the stricture. The moment he feels the resistance, he turns the stop-cock with the index-finger of the right hand (steadying the penis with the left), and propels a jet of the warmed liquid upon the strictured portion with moderate force, avoiding forcible pressure of the apparatus against the urethra. He tried this successfully in four cases, wherein he had previously failed in passing the smallest catheter.

---

### Diagnosis of True and False Ankylosis.

In *true* Ankylosis, the origins and insertions of the contracted muscles, cannot be separated by attempts to move the joint; consequently no tension of their fibres can be mechanically produced; but when *false* ankylosis exists, although extension be limited, the joint may remain capable of greater flexion, so that the resistance offered by the muscles is immediately apparent—in forming the diagnosis the history of the case may assist. Where, for instance, absolute immobility has succeeded inflammation produced by mechanical violence, you may often conclude that true ankylosis exists. The deformity resulting from *white swelling*, on the contrary, is usually incomplete ankylosis. The pain, or sense of tightness, on the flexed side of the limb, produced by manual attempts at straightening, is often an indication of the evidence of some mobility. But a much more delicate test is the production of pain on the opposite side. A completely ankylosed limb also conveys to the hand of the examiner a sensation of union throughout. The tibia and femur, in true ankylosis of the knee, give the impression of a single bone: and many of the patient's sensations in the limb are referrible to a want of elasticity, which is not absent in false ankylosis, even when no motion is *visible*.—(*Lancet, Oct.* 14, *from Dr. Little's Lectures.*)

*Ankylosis.*—Rigidity or stiffness and adhesion of a joint. The term also includes the existence of *distortion.*

*Complete or True Ankylosis.*—A perfect ossific union of the articulating surfaces, and consequent incapability of restoration to function.

In *partial* or *false* ankylosis such a degree of impediment to the motions of the joint is supposed to have taken place as materially interferes with its function, but without any union, or with merely membranous adhesion of the articular surfaces.

Simple induration of the tissues, without the existence of any ossific union, may suffice to produce complete immobility and occasion an erroneous belief in the presence of true ankylosis.

---

### OPERATION FOR FISSURE OF THE SOFT AND HARD PALATE. By J. M. WARREN, M.D.

In this case the patient was a young man, 25 years of age, with a congenital fissure of the soft and hard palate; the bones being separated quite up to the alveolar processes with a deviation to the left side. Speech extremely indistinct, and deglutition very imperfect. The soft parts were scarcely perceptible, being almost concealed in the sides of the throat from the action of the muscles. With a long double-edged knife, curved on its flat side, the operator carefully dissected up the membrane covering the hard palate, pursuing the dissection quite back to the root of the alveolar processes. By this process, the membrane seemed gradually to unfold itself, and could be easily drawn across the very wide fissure. A narrow slip was now removed from the edges of the soft palate, and with it the two halves of the uvula. By this means a continuous flap was obtained, extending back from the roots of the teeth to the edges of the velum palati.

Lastly, six sutures were introduced which obliterated the whole fissure. In three weeks the patient returned home, a firm fleshy palate being formed behind, and half the fissure in the bony palate obliterated. Some time after, one half of the latter was closed.

---

### CASE OF STRANGULATED HERNIA ON THE LEFT SIDE, AND PROTRUSION OF THE CŒCUM.

James Collier, aged two years, was discovered to have a reducible inguinal hernia on the *left* side, a few months after birth. On the 5th September last, the mother found it larger than usual, and painful. She made ineffectual attempts to reduce it, till the 8th, when Mr. Jerrard, of Honiton, was sent for. The tumor had descended to the bottom of the scrotum, and was as large as an orange. There had been no fæcal evacuation since the morning of the 5th, and there had been repeated attacks of vomiting. Mr. J. attempted the Taxis—in vain. The violent efforts and crying of the child, offered a great obstacle both to that and the operation, which was performed at 3 P.M.

"The tumor having been freely laid open, and its contents exposed, it was found to consist of the cœcum, with the appendix vermiformis, and about six inches of the ileum. The stricture was divided upwards. I now proceeded to return the protruded bowels, and here, as had been anticipated, the greatest imaginable difficulty presented itself. The child, who was very strong and muscular, continued to scream and strive in the most determined manner, so that no force, which could with safety be applied, was adequate to the returning of any portion of the bowels; and I was obliged to support them with my fingers in the best manner I could for nearly an hour, when the child became exhausted, and ceased to scream and strive, at least with much violence, and I was enabled to reduce the hernia."—*Prov. Journ.* Nov. 25, 1843.

The wound was dressed as usual, and all did well.

---

### Arch-Tourniquet.

Dr. Oke, of Southampton, has drawn the attention of the Profession to an instrument of this description.

It consists of an arch, a pad, and screw. The flanks of the arch are perforated with holes for the action of the external screw, which is worked by a short handle, as in the common tourniquet.

The pad is of the ordinary size, flat on one side, and convex on the other. Upon its flat surface there is a smooth cavity for the reception and working of the point of the screw.

Mode of application: Let the arch embrace the limb, so that one of the perforations of the flank may be exactly opposite the cavity on the flat side of the pad, previously applied over the trunk of the artery to be compressed. Then fit the external into the internal screw, and work it upon the pad till sufficient pressure be made to stop the circulation of the artery.

By this simple means any of the principal arterial trunks of the extremities may be compressed in the shortest possible time without strangulating the circumference of the limb and obscuring the operation with venous blood.

The arch is prevented from slipping by the pressure of the screw upon the pad.

These arch-tourniquets do not answer so well in practice as might be supposed. If the pressure is sufficient to arrest, or materially to control the flow of blood through the main artery, the pain is often great, and sometimes intolerable. Nor is the venous circulation so free as has been represented. The chief veins of the limb accompany the artery, and participate in its compression; the consequence is swelling of the limb. We would be far from saying that such an instrument is never useful. But its value has been over-rated.

---

### Caries of the Os Calcis successfully operated on. By Sir John Fife, Newcastle-on-Tyne.

A shoemaker, aged 24, cachectic from the effects of syphilis and mercury, was admitted into the Newcastle Infirmary, under Sir John Fife, August 24, 1843, with a sinus over the os calcis, of twelve months duration, discolouration of the integuments, and a diseased state of the bone. The following operation was successfully performed.

"An incision, nearly perpendicular, was carried from the upper and outer part of the insertion of the tendo Achillis down to the os calcis, as far as the sole of the foot. A carious surface of bone was removed by cutting forceps; the bone towards its centre appeared soft and cancellated, containing much oily matter. Lint was introduced, over which a dressing of turpentine ointment, and the latter covered by a poultice. This treatment was continued for a fortnight, and afterwards the part was dressed with simple dressings, and bandaged."

The wound healed well.—*Provincial Med. Journal,* Nov. 11, 1843.

We would observe that the removal of carious or necrosed bone, is, perhaps, too frequently neglected. The tibia is very favourably circumstanced for the operation, but the femur may be subjected to it with advantage, and, indeed, it is one of wide applicability and use. On the femur, however, it is not free from danger. We have seen a piece taken out of the popliteal artery, where it is just entering the ham, by the trephine. Fatal hæmorrhage was the result, amputation being resorted to ineffectually.

---

### NEW TRUSS.

At the Sheffield Medical Society, Mr. Overend exhibited a truss with a spiral spring in the centre of the pad, invented by a mechanic, resident in the neighbourhood of the town, who has for several years been affected with *direct* inguinal hernia. He had experienced much more relief by wearing a truss of this description than from any he had previously worn.—*Prov. Medical Journ.* Nov. 11, 1843.

---

### NEW MODE OF REDUCING A LUXATION OF THE HEAD OF THE FEMUR ON THE DORSUM ILII.

A young man, not very powerful, met with a dislocation of the head of the left femur on the dorsum of the ilium, occasioned by the fall of a bale of cochineal upon his back as he was stooping. Reduction was at first attempted by the usual method of extension, with towels as he lay upon the right side. Then he was put on his back, and reduction again attempted in vain. Another plan was now tried by Mr. Clark, surgeon of the South Hants Infirmary, under whose care the patient was.

"I now flexed the knee to a right angle, which raised the thigh to an angle of about forty-five degrees with the bed, perhaps near a right angle with regard to the pelvis; and, by bringing the foot in contact with the other leg, while the knee was sustained in a perpendicular direction, it is manifest that the femur was rotated, and the trochanter major thrown outwards, the head alone then resting on the ileum behind the acetabulum, which cavity, with the axis of the femur through its head, neck and shaft, were now nearly on the same plane; in this relative position of the parts, by throwing the limb outwards, the head of the bone, it is fair to infer, would start forwards to its natural situation; and such was the gratifying termination of the case, with no more force than was necessary to abduct the limbs. The pelvis still fixed to the right side of the bed, admitted of no change; the heel rested on the bed, as above, while I stood on the left side of the patient, with my right hand on the hip-joint, and my left holding the knee; thus the reduction was readily and audibly effected as I drew the limb towards me."—*Prov. Med. Journ.* Dec. 9, 1843.

### THERAPEUTICS.

### Treatment of Hydrophobia by Compression of the Carotids.

Dr. Allier states that, in 1837, while attending a servant attacked with confirmed hydrophobia, he was forcibly affected by the total impotence of medicine.

Recollecting then the efficacy of compression of the carotids in cases of epilepsy, he compressed simultaneously these two arteries at the commencement of a convulsive paroxysm. In an instant the convulsions ceased, and the patient fell into a kind of fainting fit. Alarmed at this, the family refused to allow the doctor to continue the methodical employment of this powerful treatment. The hydrophobic symptoms again showed with renewed violence, and the patient died on the following day, forty-eight hours from the commencement of the attack.

Dr. Allier remarks, that the compression of the carotids has not yet been employed, to his knowledge, in this terrible affection; its effects were sufficiently evident in the case above related to render it important to ascertain afresh its definitive influence on rabies. It is true that, by the employment of this measure, you do not attack the evil at its source, you do not destroy the *intoxication*; but, perhaps, in annihilating thus, by compression of the carotids, the deadly effects of this poison, namely the convulsions, the fatal termination of this frightful malady might be prevented; since these convulsions are the cause of death either by asphyxia, or by the exhaustion of the nervous system.—*Clinique des Hôpitaux des Enfans.*

### Dr. Wigan on Tinea Capitis.

*Mode of Treatment proposed by Dr. Wigan.*—The head is to be shaved carefully twice. The remedy proposed is Beaufoy's *concentrated acetic acid*—as a preliminary, the acid diluted with three times its weight of water is to be used as a test or detector acid. On the application of this, a number of spots previously looking healthy become red patches—then with a piece of fine sponge tied to the end of a stick, or held in a pair of silver sugar-tongs, imbue each spot thoroughly with the strong acid for three or four minutes, and the business is done, one application being enough. The crust grows up with the hair—this should be removed as soon as a pair of fine scissors can be introduced beneath it.—*Medical Gazette*, Sept. 15, 1843.

### Varus Mentagra and Gutta Rosea, the Sycosis Menti and Acne of Willan, treated with Sulphate of Iron Externally. By W. Dauverger.

The sulphate of iron is used in solution, either by bathing the part affected, or by applying linen dipped in it, or by sprinkling the ulcerated parts of the mentagra with a mixture of charcoal and sulphate of iron. The following are the formulæ employed by him.

No. 1.—Sulphate of iron 25 grammes; Distilled water 200 grammes—dissolve.

No. 2.—Double the strength of the above.

No. 3.—*Ferro-carbonic powder.*—Sulphate of iron 10 grammes; Charcoal 35 grammes—powder and mix.

The inflammatory symptoms are treated with emollients. Then the patient is to bathe the part twice a day with two glasses of warm water containing one or two spoonfuls of No. 1. A quarter of an hour after, he prescribes a local bath of an emollient decoction; and afterwards, if possible, a poultice of the same kind. If no improvement take place, he goes to No. 2. General means of treatment are used at the same time.—*Gazette Medicale*, Sept. 9, 1843.

---

### OBSERVATIONS ON SPERMATORRHŒA. By W. H. RANKING, M.D. Cantab.

Spermatorrhœa is sometimes the consequence of continence carried to excess. The involuntary emissions, which constitute a really morbid phenomenon, are, however, usually traceable to a very different cause.

*Causes.—(Predisposing:)* A congenitally feeble state of the sexual organs, by which they are rendered easily susceptible of injury from over-excitement: this state is indicated by congenital phymosis, varicocele, and incontinence of urine. *(Exciting:)* Over-excitement of the organs, gonorrhœal inflammation of the urethra, affections of the rectum, constipation and ascarides, presence of sebaceous matter under the prepuce, and continence.

The *Modus Operandi* of these causes is sufficiently obvious.

*Symptoms.*—After abuse of the genital organs the patient finds himself infested with seminal emissions during sleep, which are at first accompanied by erections, but these erections soon cease—in sexual intercourse at this time he experiences more than usual difficulty in consummating the act; there is incomplete erection —ejaculation often difficult and painful. As the disease advances, the emissions increase in quantity and frequency, the patient being made conscious of them only by the sense of weakness on awaking. In sexual intercourse the ejaculation becomes more hurried; the mere sight or touch of a female will cause it. The mind becomes enfeebled, as well as depressed—cerebral and thoracic symptoms now occur—giddiness, noises in the ears, palpitation and cough. Digestion is impaired—bowels costive. Micturition frequent during the night—penis flaccid and inelastic—scrotum pendulous—testicles soft and tender to the touch. Urine turbid and nauseous to the smell, the turbidness appearing only towards the end—the emptying the bladder and evacuation of the bowels are accompanied by a seminal discharge. It is important to remark that the tendency of every case of morbid nocturnal emission is to become diurnal, if it be unchecked. Hence the absence of nocturnal discharge is no proof that the disease has vanished.

*Morbid Anatomy.*—1. Orifices of the *Seminal ducts*, or those ducts themselves dilated, or as it were dissected out by suppuration of the prostate—ducts cartilaginous or ossified. 2. *Vesiculæ Seminales* filled with pus, or with tubercular matter, mixed with ill-elaborated semen. 3. *Vasa deferentia* tortuous and irregularly dilated. 4. Testicles soft and white, and diminished. Hence the essential lesion of spermatorrhœa is chronic inflammation of the parts concerned in the formation, conveyance, and expulsion of the seminal fluid.

*Treatment.*—First ascertain the exciting cause—if ascarides in the rectum are present, expel them in the usual way. When the complaint depends on stricture of the rectum, fissure of the anus, or hæmorrhoids, employ the appropriate surgical treatment—the sebaceous matter behind the prepuce is to be cleansed away. When the disease depends on general debility, and on an atonic condition of the spermatic vessels, cold bathing and douches on the parts will be necessary. Mineral tonics also—the best is the tincture of the sesqui-chloride of iron. When the disease depends on undue exertion of the sexual organs, the excess must be relinquished, sexual intercourse prohibited—no stimulating food or drinks. Nitrate of silver is to be applied to the affected portions of the urethra; one application generally suffices. Mode of operating:—a common bougie or catheter is to be passed first, and the length is to be noted at which the urine begins to flow—an inch short of this gives the site of the membranous part. The armed instrument is then to be passed to the same length, and the caustic made to revolve, by twisting the instrument for the space of an inch and a half, when it is to be drawn within the bougie and extracted;—if there is much pain or ardor urinæ, a hip bath may be used—leeches will occasionally be found useful—patient should lie on a hard mattrass, and a cold enema with 20 drops of laudanum at going to bed will assist in preventing the discharge.—*Lancet*, Oct. 14, 1843.

Mr. James Douglas, Lecturer on Anatomy in Glasgow, proposed, in the Medical Gazette, Sept. 29, 1843, to substitute a solution of sugar of lead with opium mixed with mucilage, as an injection, for the nitrate of silver, he having found it very successful in several cases.

---

### Naphtha in Phthisis.

There has been a sort of controversy going on lately on the question if naptha does or does not cure phthisis. Now probability would say no—some gentlemen say yes. Those who like facts have been favoured with the following from Dr. Ranking of Bury St. Edmunds, who has published them in the Lancet. He has given it in eight cases, three females and five males. Of these, three are already dead, the remainder are still alive, but dying gradually. In five of these cases there were unequivocal signs of cavities in one or both lungs, with emaciation and copious purulent expectoration. In the other three, the disease was not so far advanced: but, besides the signs of tubercular deposit, crepitous and submucous râles, with the appearance of pus in the sputa, indicated that softening had commenced. In none was the most trivial benefit perceptible; neither was the perspiration checked, as it is stated to be, as if by magic; nor were the cough and expectoration diminished. In two instances nausea was complained of, and every patient, without exception, implored its omission.

---

### Nitrate of Silver to prevent Bed-sores, and for Burns.

Mr. Jackson, in a Paper read before the Sheffield Medical Society, has recommended the nitrate of silver for the prevention, or the cure of bed-sores.

The form which he uses is in the proportion of ten grains of the nitrate of silver to one ounce of water, applied by means of a camel-hair brush over every part exhibiting the slightest appearance of inflammation, two or three times a day, until the skin has become blackened; afterwards only occasionally.

Mr. Jackson lauds it in the treatment of burns and scalds. He instances several cases of superficial burns in children in which he found that in a very short time after its application the pain ceased, and vesication was totally prevented.

In the deeper burns he uses it, not that he finds that it can produce any effect upon the charred parts, but that, as Mr. Higginbottom has said, he finds the superficial burn healed, and the extent consequently circumscribed.—*Provincial Medical Journal.*

For our own parts, we question whether *any* applications will answer in the long run, either for preventing or for curing bed-sores, unless pressure is removed—and, if it be removed in time, whether any are necessary. Give us a good piece of "buffalo's skin" with or without a hole in it, and attention to cleanliness, and we will make a present of all the drugs in the Pharmacopœia to our adversary. Prevent pressure then, and it *may* be prevented, and the bed-sores will take care of themselves.

### CURE OF EPISTAXIS.

According to Dr. Negrier, one way of doing this is to hold up one or both arms, and close the nose at the same time. Simple, if successful. We have no doubt that, as in most cases, bleeding at the nose stops of itself, holding up the arms would in such, be certain to answer.

### MIDWIFERY.

### ON THE TREATMENT OF PUERPERAL CONVULSIONS BEFORE THE FULL TERM OF UTERO-GESTATION. By S. HARRIS, M.D. of Clarkesville, U.S.

The object of the author is to call the attention of the profession to a material point in the treatment of puerperal convulsions, when the ordinary remedies fail, and it becomes necessary to resort to delivery, at a time that the os uteri is found undilated, and undilatable by gentle means. The general rule is, that if the os tincæ is not dilated, or dilatable by easy means, no forcible entry into the uterus must be made under any circumstances. This is the rule laid down by the highest authorities in midwifery. The great difficulty is, when a case occurs in which delivery or death is the only alternative. Must we then, says Dr. Harris, quietly seat ourselves, and witness the certain triumph of this terrible disorder? Must the case be given up to nature, or are we not justifiable in making a forcible entry into the uterus, and extracting its contents? He expresses himself decidedly in favour of this last painful resource; he even considers it practicable in most cases, unless in the very early stages of pregnancy. He cites the case of a strong healthy young woman of about 16 years of age; in the fifth month of her pregnancy she was attacked with puerperal convulsions. All the ordinary means had been employed without any good effect. When he was called in, he found her in a state of total insensibility. Breathing laboured and stertorous—pulse weak and fluttering, sometimes imperceptible—no uterine action—os tincæ almost entirely closed, hard and unyielding—paroxysms recurred with unabated fury. Forcible delivery was accomplished. Blisters and warm applications were applied to the extremities, which were cold and clammy. She had two or three fits after delivery. She remained comatose for nearly 24 hours after, frequently without any pulse at the wrist. By proper care, however, she was soon restored to perfect health, and since then she has been pregnant three or four times. He cites another case somewhat similar, wherein he succeeded equally well in extracting the child without doing any serious injury to the uterus; but in which the patient eventually died, the forced delivery having, as he thinks, been too long delayed. The author of this paper acknowledges that it is only as a *pis-aller* he can recommend this course of forcibly entering the uterus and delivering the fœtus. He ventures to affirm, that if timely resorted to, it will, under the guidance of reason and the promptings of ingenuity, result very often in the preservation of human life.—*American Journal of the Medical Sciences*, July, 1843.

### Obstetrical Auscultation. By H. Van Arsdale, M.D. of New York.

Among the sounds which obstetrical auscultation reveals, are two—the *uterine souffle*, and the *pulsations of the fœtal heart;* the former being an *uncertain*, and the latter a certain and positive sign of pregnancy. The uterine souffle is dependent on the arterial circulation of the mother; it is perfectly coincident with the mother's pulse. Laennec erroneously thought that this souffle was produced in the principal artery nourishing the placenta, and others were equally in error in supposing it to be produced in the iliac artery. The author, from his inquiries into the the nature and value of the uterine souffle, lays down the following propositions:—

1. It differs entirely from the various arterial souffles: freedom from any shock, proximity to the ear, and want of uniformity are its peculiar characters.

2. Its presence is not an invariable and sure proof of the existence of pregnancy; it is sometimes detected in fibrous diseases of the uterus.

3. It is produced in the uterine arteries, and the explanation of its irregularities, intermittences and change of place, may be found in the difference of the calibre of the arteries on penetrating the womb, and also in the change of position of the fœtus in utero.

4. The point where the souffle is perceived, does not always correspond with the insertion of the placenta.

5. The period of its appearance is not constant. It is very difficult to discover before the end of the third month; but it is rarely absent at the end of the fourth, and continues to increase in intensity till the end of the sixth month. From this period it varies rather in its nature than in its intensity.

6. It is found most often in the lateral portions of the uterus, a short distance above the crural arches; but it is sometimes heard along the median line.

7. It is of no consequence in the diagnosis of cases of double pregnancy, of diseases of the uterus or placenta, or of the life or death of the fœtus.

The double pulsations, or the pulsations of the fœtal heart, are far the more important of the two sounds discoverable by auscultation. From his observations and researches on this important sign, the following propositions have been laid down:—

1. These double pulsations are a sure sign of the existence of pregnancy and of the life of the fœtus.

2. The variations in the circulation of the mother have no effect on that of the child.

3. The *increase* in the number of the double pulsations is unimportant, as being of rare occurrence, and of short duration—whilst, on the contrary, a *decrease* in the number, during parturition, and especially in a protracted labour, announces danger to the child, and indicates that the labour should be terminated by instruments, by version, &c.

4. These pulsations are not to be expected before the completion of the first half of pregnancy. The average number of beats is 135 in a minute, and they continue to increase in intensity from the moment when first heard until parturition.

5. Cases of double pregnancy may be diagnosed from hearing the pulsations of two distinct fœtal hearts, and these pulsations usually differ in number from eight to fifteen.

6. The detection of these pulsations is of the first importance in cases of extra-uterine pregnancy, since, if they were not discovered, the case may be mistaken for a fibrous tumor of the ovaries, or a simple cyst, and be treated as such.

7. They are discovered at a point corresponding with the dorsal præcordial region of the fœtus.

8. To discover the presentation, we have to draw an imaginary *horizontal* line, dividing the uterus into two equal parts. When the head presents, the pulsations are heard in the lower half; and when the pelvis presents, they are heard in the upper half.

9. To discover the position, we have to draw an ideal *vertical* line, bisecting the preceding one. When the back of the child is turned towards the right side, the pulsations are heard in the right half; and when turned to the left, they will be heard in the left half.

Lastly, the total absence of these pulsations should invariably deter us from performing the Cæsarian operation, or, indeed, from doing any thing which may prove injurious to the mother, in the vain hope of aiding the child, which we are now bound to believe to be no longer alive.— (*New York Journal of Medicine,* Sept. 1843.)

---

### ON INFLAMMATION AND ABSCESS OF THE UTERINE APPENDAGES. By FLEETWOOD CHURCHILL, M.D.

The author of this paper is of opinion that many females have owed the delicate health in which they have remained for some time after their confinement to inflammation and suppuration taking place in the uterine appendages, that is, in the Fallopian tubes, the broad ligaments, or the ovaries; this condition of the parts has been unnoticed in consequence of the obscurity of the symptoms. He illustrates the disease by citing twenty-three cases of it collected by him from various sources. The first of these cases was that of a woman 44 years of age, who had had five children, the youngest being but two years of age. For some time past she had felt pain in the inguinal regions and above the pubis, which she attributed to pregnancy. Shortly after consulting the Doctor the pain increased, and she felt something give way to the left of the pubis, and immediately after a quantity of puriform matter was passed by the rectum, from whence a sanguineo-purulent discharge continued for a week, and then ceased, and she recovered. After a few weeks she had a return of the pain, followed by the discharge, which ceased again after a week or two. During the week preceding the discharge, she suffered from severe dragging pain in the inguinal regions, perspirations, loss of appetite, dysuria and tenesmus, all which symptoms disappeared after the matter was evacuated. The remedies employed were leeches and poultices to the seat of the pain; small doses of calomel and James's powder, and an occasional aperient. Another case was that of a woman aged 40, who was delivered by the forceps of a living child. She had been long in the first stage of labour, and the cervix uteri was torn off. Before assistance could be rendered, the perinæum was lacerated, and she had a tedious recovery, during which she had an attack of hysteritis, which was subdued by the ordinary means—in some time after she had an attack of peritonitis, and another attack in a month after. The cause of these sudden attacks was found to be a tumor near the right iliac region as large as a goose egg, and very tender on pressure. Two days after the detection of this tumor a large quantity of matter escaped from the vagina and rectum, and the tumor greatly diminished in size, and lost its tenderness. The discharge occasionally stopped for a day, and then returned, until all tumefaction had disappeared. She gradually recovered strength. In this case the escape of matter into the peritoneum was the cause of the two attacks of the peritonitis.

This inflammation of the uterine appendages may occur in an acute or chronic form. In the former it constitutes one of the varieties of puerperal fever. It is to the chronic form of the disease that Dr. Churchill has more particularly directed his attention. The *causes* of the attack are not easily assigned; it may follow blows, falls, or a fright; it more frequently results from cold. It is sometimes attributed to the suppression of the milk or the lochia. The mode of invasion varies a good deal: sometimes there are few, if any, preliminary symptoms; uneasiness, perhaps, in one iliac region, and on placing her hand on the spot the patient detects a tumor. With respect to the *symptoms*, there is a distinct tumor—this may be found completely above Poupart's ligament, above the linea ileo-pectinea, sometimes occupying one iliac fossa entirely, and even extending upwards nearly to the umbilicus, and forwards to the linea alba—or it may be seated more deeply in the pelvis, just reaching to Poupart's ligament, protruding the groin, and from its fixedness giving the impression of its being firmly connected with these parts. The tumor is hard as a stone until suppuration commences, and equally tender on pressure. Stings of pain radiate in all directions from the tumor—when the tumor is situate in the pelvis and groin, the pain extends across that cavity, down to the anus, back, and down the thigh—standing upright is very difficult and painful, as also walking—there is tenesmus, and a desire to make water frequently. Fever is also present. The *terminations* may be resolution, or—abscess. The matter of this abscess may escape—*a.* externally through the abdominal parietes—*b.* into the peritoneum, causing peritonitis —*c.* into the vagina—*d.* into the bladder or rectum—*e.* into the surrounding cellular tissue—*f.* the extent of the disease, or the secondary affections caused by it, may prove fatal after an indefinite time. With respect to *treatment*, the indications are—1st. To procure resolution of the tumor; 2. To promote suppuration and evacuation of the matter. The former is best fulfilled by the application of leeches to the tumor, to be followed by bran-poultices, and repeated, if necessary. Fomentations and a hip-bath will also assist. If, however, suppuration take place, an opening should be made into the abscess, when it is possible, so as to decide the course the matter is to take—the best situation for the opening is through the abdominal parietes—the next through the vagina.—(*The Dublin Journal of Medical Science*, Sept. 1843.)

---

### On Puerperal Convulsions. By Charles Halpin, M.D. Observations on Puerperal Convulsions. By Robert Johns, M.D.

Puerperal convulsions have been divided into three species—epileptical, apoplectical, and hysterical; the epileptical is the most frequent—they may occur either during the latter months of gestation, during labour, or after delivery. An attack of convulsions may set in at a time, when, from the favorable condition of the woman, we have least reason to expect it. Every thing may be progressing to our satisfaction, when suddenly, and without a moment's warning, a scream, or some stertorous or hissing sound from the woman, attracts our attention. On turning round, we find her in a state of insensibility; if she has been standing or sitting previously, she has fallen to the ground—body violently agitated—entire muscular system forcibly and irregularly contracted—hands firmly clenched—arms and limbs flung wildly round, or rigidly extended—head drawn violently backward, and neck twisted so, that frequently the face is turned toward either shoulder; eye-balls projected, either fixed with an appalling stare, or rolling wildly, and ready to start from their sockets; cheeks and lips red or livid—tongue protruded—mouth filled with frothy mucus—vessels of head and neck swollen and turgid—breathing stertorous, or accompanied with a peculiar hissing sound—pulse either full, slow, and soft, or very rapid, and frequently intermittent.

Dr. Halpin lays down the following propositions, as conclusions fairly deducible from his observations.

Puerperal convulsions occur most frequently in first pregnancies; the ratio being six in seven. Among the predisposing causes, the age of the patient seems to have much influence. In one-fourth of the recorded cases the women were above 28 years old. There is no decided premonitory symptom invariably present. The presentation is almost invariably natural. They are always attended with danger both to mother and child. One-fourth of the mothers and two-thirds of the children perish. As to treatment, copious blood-letting, early resorted to, and rapidly effected, is of the first importance. Active purgatives should be given, either by the mouth or in injections, until the stomach and bowels are thoroughly cleansed of offensive matter. Should these measures fail, the uterus should be emptied of its contents as early as the state of the parts will admit of its being done *without violence*. The natural efforts are frequently sufficient to effect the delivery. Where interference is required, the forceps or vectis are to be preferred in many cases to turning, or the perforator, as turning the child is attended with great danger to the mother, as five out of seven of those on whom it has been practised have died.

Contrary to the opinion advanced by Dr. Halpin, viz. that there was no premonitory symptom in this affection, by a proper attention to which the attack may be prevented, Dr. Johns lays it down, that if, during the last months of pregnancy, the superior parts of the body, as the hands and arms, the neck and face, should become œdematous, the case will require very close and attentive examination; for if, in combination with this symptom, there exist headache, weight, or giddiness in the head, ringing in the ears, a temporary loss of vision, severe pain in the stomach with flushed face, there will be risk of convulsions—a risk that will be converted into certainty, if—1, the woman is pregnant for the first time, or had suffered similarly in former pregnancies; 2, where the head of the child presents, as in ordinary labours; and 3, where the woman is of a full and plethoric habit.—*Dublin Journal of Medical Science*, Sept. 1843.

### PHYSIOLOGY.

### ABSTRACT OF A REPORT BY MR. WHARTON JONES ON THE OVUM OF MAN AND THE MAMMIFERA, BEFORE AND AFTER FECUNDATION.

*Before Fecundation.*—The *Graafian follicles,* formerly supposed to be the ovarian ova, have been now proved to be merely the containing capsules of the real ova. The inner surface of the Graafian follicle is lined by a membraniform layer of nucleated cells, 1.2000th of an inch in diameter, called by Baer the *membrana granulosa.* At its surface this membrane is thicker than elsewhere, presenting there a greater number of cells, erroneously called by Baer *discus proligerus.* In the central part of it the ovum is imbedded, as an opake white speck.

*Ovum and Yolk.*—The granular mass in the centre of the ovum is analogous to the yolk of the bird's egg; the broad transparent ring or *zona pellucida* surrounding it is acknowledged to be the optical expression of the circumferential doubling of a thick transparent membrane which encloses the yolk. The yolk consists of granules, the larger about 1.5000th of an inch in diameter, held together by a clear, viscid substance—the density of the yolk increases with its maturity. The *germinal vesicle* of mammifera was first discovered in the ovum of the rabbit—in such ova it is about 1.600th of an inch in diameter. Under a compound microscope it is seen as a simple cell—a transparent membranous wall with perfectly clear contents. At one side of it is a small dark, round spot, called by Wagner the "germinal spot," and is of general occurrence throughout the animal series. The germinal spot of the mammiferous ovum is a disc

1.3000th of an inch in diameter, composed of a finely granulated substance, strongly refractive of light, and attached to one part of the inner surface of the wall of the germinal vesicle, which it causes to be more prominent there than elsewhere. According to Wharton Jones the germinal vesicle and its spot stand in the relation to each other of cell and nucleus.

*Fœcundation.*—Actual contact of the seminal fluid with the ova takes place in many of the lower animals; and in the mammifera, it has been shewn that "post coitum all the surfaces over which the ovum or ova must pass, from the ovary to the uterus, become spread over with spermatozoa." Where does this contact take place? Bischoff and Barry believe that it occurs in the ovary. At the moment of ejaculation the semen is received directly into the uterus—it may reach the ovary partly by diffusion through the mucus in the genital passages, partly by the movement of the spermatozoa, which, according to Henle, can move one inch in seven minutes and half. Another means to promote the progress of the seminal fluid towards the ovaries is quick and progressive, but not peristaltic, contractions, of the uterus and Fallopian tubes from the vagina onwards, which have been observed in bitches and rabbits post coitum.

*Ovum before leaving the Ovary.*—In the rabbit from nine to ten hours elapse *post coitum,* before the Graafian follicles burst—in other animals the time is variable. The ovum *having passed into the Fallopian tube,* acquires there a deposit of a thick, gelatinous matter. This investment increases in thickness according as the ova proceed in the tubes. "It has a stratified texture, and deserves the designation of white of egg." In their passage through the Fallopian tubes, the ova (of the rabbit) become double or more than double the size of ovarian ova. The changes in the yolk during this period are very interesting: it suffers division and subdivision after fecundation. The germinal vesicle has disappeared, but is replaced by two other vesicles or cells, each having its nucleus. Each of these cells is resolved into two other cells, and so on, until the germ consists of a mulberry-like object, the cells of which are so numerous as not to be countable. (The very great complexity of the process here obliges us to refer our readers to Mr. Jones' Report itself—or to Willis' Translation of Wagner's Physiology.) The ova, on entering the Fallopian tubes, are surrounded by cells of the disc and membrana *granulosa* of the ovary; but these soon begin to be absorbed, and in the first or upper half of the tube they have wholly disappeared; that is, where the ovum is invested in the tube with a gelatinous layer. The ova in the rabbit reach the uterus about the third or fourth day after coitus—with respect to the human ovum, the time has not been ascertained.

*The Ovum in the Uterus, the Amnion, and the Blastodermic Vesicle.*—When the ovum has reached the uterus, by which time the ovum has increased in diameter from one-fifth to half a Paris line, a layer of vesicles, similar to those constituting the mulberry-like structure, now appears lining the vitellary membrane or zone: where this layer and the mulberry-like object are in contact, a new membrane is generated, considered by Barry as the future *amnion.* In the interior of the mulberry-like structure there is now a large vesicle, in the centre of which is a spherical body, which seems to be the *true germ.* Thus, according to Barry, the rudimental embryo is the nucleus of a cell. On the fifth day, *post coitum,* in the blastodermic vesicle is observed an opake spot, the *arca germinativa, cumulus germinativus,* &c. of authors. In ova of the 6th day, small inequalities are seen in the ovum, which are soon recognised as the villous processes of the *chorion,* a membrane formed partially at least from the external membrane of the ovum. From about the beginning of the ninth day the ovum has contracted an intimate connexion with the uterine mucous membrane. The latter is raised into minute folds and villi, covered by epithelium, which adheres closely to the external membrane of the ovum. In ova of the rabbit, half an inch in diameter, the blastodermic vesicle has been found to lie free within the external membrane. The arca germinativa is on the side opposite the insertion of the mesometrium. In a few hours the vesicle begins to be united by its animal layer to the external membrane, and then indirectly to the uterus. The blastodermic vesicle now changes forms. "It is no longer round, but first oval and then pear-shaped, its long axis always falling on the transverse axis of the somewhat oval ovum. In all these forms it is composed of the *area opaca*, enclosing a clear spore, *area pellucida*. These differences are owing to the different accumulation of cell-materials, especially in the animal layer; the cells appear to pass from the centre to the periphery, leaving the centre transparent. In the long axis of the area pellucida there is seen *a clear streak;* with this commences the first trace of the *proper embryo*. This usually occurs on the eighth or ninth day post coitum. Around and on either side of this streak formative opake matter collects, chiefly in the *animal* (serous) layer. The bright streak is a groove in that layer; at the end of it, corresponding with the broad end of the pear-shaped area, its two edges pass into each other by a small arch, whilst at the other end they unite in a point. The former is the head end, the latter the tail end, of the future embryo. The two accumulations of formative matter on either side of the groove Reichart considers to be the original halves of the nervous system; Bischoff considers them the *original basis of the body of the embryo*.

The *area opaca* having extended itself, and again become round, and the *area pellucida* and the basis of the body of the embryo having become fiddle-shaped, that portion of the formative matter which immediately constitutes the two sides of the primitive groove, rises up in the form of "dorsal plates." These, meeting in the middle over the groove, convert it into the canal, in which are formed the central organs of the nervous system. Subsequently the circumferential part of the same formative matter becoming thickened, and bending downwards and inwards, constitutes "visceral plates."

Hence the development of the embryo of the mammifera is seen to be analogous to that of the embryo of the bird. So also is the farther development. The "dorsal plates" having closed in, the formation of the nervous centres having commenced, and the head end of the dorsal canal having become dilated, the embryo bends forward on itself, and an elevation of its anterior, and, in a less degree, of its posterior end, takes place from and above the plane of the blastodermic vesicle, so that the embryo is, as it were, constricted off from it. The margins of the "visceral plates" all round approach each other, and inclose the "visceral cavity," and gradually these plates also separate off from the blastodermic vesicle behind. The vegetative follows the direction given by the animal layer in the process; and above and below it is drawn within the visceral tube now in process of formation; but from the points of constriction, the vegetative layer is continued over the head and tail ends of the embryo, the coverings it thus forms, being the head and tail *involucra*. For the description of the formation of the amnion and chorion we must refer to the original Report. About the time the amnion begins to be formed, the vascular system first appears in the form of a *heart-canal*, in the anterior wall of the head-end of the embryo, where it is continued into the blastodermic vesicle: a *vena terminalis* lines the periphery of the area opaca; and throughout the latter a vascular network may be traced between the two. The amnion having been formed, the development of the intestinal canal goes on; and during this the embryo, by development and approximation of the visceral plates, becomes more and more separated by constriction from the vegetative and vascular layers of the blastodermic vesicle, which now constitutes the *umbilical vesicle*. The vessels of the vascular layer, consisting of one or two veins, carrying blood from the yolk to the embryo, and an artery proceeding from it, are called omphalo-mesenteric. The canal, to which communication between the intestine and umbilical vesicle is now reduced, is the ductus *omphalo-mesentericus* or *vitello-intestinalis*. During the formation of the umbilical vesicle, a small and very vascular vesicle, at first round, afterwards pear-shaped, sprouts out from the lower free end of the embryo. This is the *allantois,* a structure of the utmost importance in the further development of the embryo and ovum. The development of this differs much in the different orders of the mammalia. Its early disappearance or obscurity in our species formerly led to the erroneous idea, that no allantois exists in the human ovum. M. Serres, at a sitting of the Paris Academy last June, brought forward certain views on the origin of the allantois in the human ovum. The conclusions he arrives at from his investigations are:—that the human allantois is, as that of the rodentia, a pyriform vesicle, at first independent of the other membranes of the ovum; that it finally joins with the chorion, its vessels combining with the villi of the latter in originating the placenta; and that the isolated condition of the allantois in the human subject is limited to the period of about between 15 to 25 days from conception.—*Lancet,* Nov. 25, and Dec. 2, 1843.

---

### MORALITY *versus* PHYSIOLOGY.

There has been a good deal of ink shed within this month or two on impotence, seminal weakness, and so forth. One gentleman laments pathetically enough, and not without reason, that the quacks have got possession of the subject. A student tells us that the regular lecturers seem to have *tabooed* it, but hopes for a new era and for better times. The main battle, however, has turned on the moral point of the question. One party contends that Nature meant men to have connexion with the female sex, and that prolonged continence or masturbation, leads to an irritable condition of the generative organs, nocturnal emissions, "spermatorrhœa," and the ills that follow them.

Dr. Bull, on the other hand, protests against this doctrine as at once unphilosophical and irreligious, and declares with warmth that "*the laws of physiology are never at variance with those of morality.*"

How far this abstract position may be sound, we will not take upon us to determine. But this we will say—that if morality means continence, the laws of physiology are *not* in its favour. Ask the opinions of men in practice. How many instances do they not see of health damaged by forced chastity in the female sex—of health restored by the mere fact of marriage. Amongst men, fewer cases of disorder or disease induced by abstinence from sexual intercourse present themselves, because men do not and will not restrain their inclinations to the extent to which women *must.* But the great experiment of the Church of Rome has surely settled the matter. The celibacy of its clergy has been a signal failure in a moral sense, however it may have served the political objects of the Papacy. Nature has been too strong for the fetters of priestly policy or of religion, and impaired health, or a fanatical asceticism upon one side, with every species of excess upon the other, have been, in the gross, the products of the system.

If we descend to private and personal observation, we find corroborations of the same fact. Men, who, in early life, put an absolute check on their passions, are found on the whole to suffer from an irritable state of testis, headaches, malaise, &c., and from nocturnal emissions. How rarely do we see these in married men! In short, the morality of *Nature* and of *Physiology,* if such morality there be, points not to continence, but marriage, and the condition which best realizes *every* requisite appears to be *monogamy.*

---

### The Physiological Effects of Highly Condensed Air on the Human Body. By W. Detmold.

In the Archives for Mineralogy, Geology, and Mining, an article occurs giving a description of an ingenious contrivance in mining, where one element is made use of to resist another, and where human skill comes out victorious from the contest of the two elements. The object of the contrivance was to keep out the influx of water from a mining shaft by means of condensed air. The part of the article interesting to the medical man is the effect produced on animal life, that is upon the miners at work in the condensed atmosphere. The first phenomenon observed when the men entered into the condensed air, was a more or less severe pain in the ears. The pain commenced immediately on entering into the condensed atmosphere, and ceased as soon as an equilibrium was established between the condensed air and the air contained in the interior of the ear. In some cases the pain was scarcely perceptible. Another singular observation made by the workmen was, that no body was able to whistle in the condensed air. This the writer would explain in this way; whistling consists in sending a stream of condensed air through a small aperture in the lips, whereby sound is produced; but under a pressure of three atmospheres the air is already so condensed that the ordinary effort which the muscles of the cheek and lips and the respiratory muscles are accustomed to make for the purpose of whistling, is not sufficient to compress the air any further. Another fact observed was, that in this condensed air *every body speaks through the nose;* that is, in correct language, the sound becomes nasal. This may arise from the pressure of the external air condensed on the nose being greater than that of the air passing through the interior of the nose, which air suffers some rarefaction by the animal heat. Another effect observed was that the workmen, in ascending the shaft filled with condensed air, did not become short of breath to the same degree, as they would have done in the free air—this is explained by the greater quantity of oxygen contained in a given volume of the condensed air than of the ordinary air, for which reason the lungs require a smaller volume of air to decarbonize the blood; hence the action of the respiratory muscles need not be so energetic. Might not this fact be made available in certain pulmonary affections, as asthma? Another extraordinary effect observed was, that a man who had been deaf, always heard better in the condensed air than any of the other miners, who were not deaf. How is this to be explained?—(*New York Journal of Medicine, Sept.* 1843.)

---

### Remarks on Fatty Urine, by Golding Bird, A.M. M.D.

April 22. Dr. G. Bird visited a Mrs. T. whilst still in bed. She was extremely fat, 35 years of age, and a mother. She was in perfect health—complained only of the occasional milky state of her urine, as possibly indicating some threatening ailment. She had been accustomed for several years to pass milky urine, especially during pregnancy. Frequently the urine, though not milky, had gelatinized on cooling. The appearance of this milky urine was not constant. This state most frequently appeared, when she first voided urine on arising from bed. It had become most frequent since she began to grow fat. The visit of Dr. G. B. was at 2 P.M. Three specimens of urine were shewn him as having been passed since morning. The first was like ordinary urine, containing much pinkish urate of ammonia, which disappeared by heat—acid—not coagulable; no albumen. The second was pale as water, sub-acid, and on heating clouds formed in it from coagulation of albumen. The third had a natural appearance and no albumen. She stated that this was the first time she ceased to pass milky urine for the last three weeks. Dr. B. introduced a catheter and drew off a pint of fluid, having the odour, colour, &c. of hot milk and water—sp. grav. 1010—slightly acid—by repose a cream-like colour formed on its surface, the lower portion being nearly transparent. The following are the chemical characters of this milk-like urine.

A. A large and tremulous coagulum of albumen formed by heat, becoming firmer and more solid on raising temperature to boiling. B. On agitating about four ounces of the urine with half an ounce of pure ether, and setting the mixture aside in a well closed bottle, the mixture on the next day lost its opacity and presented three well defined layers—the lowest (the great bulk) was transparent urine—on its surface rested a transparent firm coagulum of fibrine, of a pale yellowish colour. The upper layer was an ethereal solution of fatty matter. When the latter was allowed to evaporate, a large proportion of yellow fat, like butter, was left. On examining the urine under the microscope no appearance of oil globules, blood-discs, or pus granules could be detected. This is directly opposed to the statements of some continental observers on the subject of fatty urine. Dr. G. B. considered this case to be one in which an excess of fat, not capable of any other appropriation, escaped by the kidneys in the form of an emulsion with the spontaneously coagulable albumen of the blood.—(*Medical Gazette,* Oct. 27, 1843).

### MATERIA MEDICA AND PHARMACY.

### On Yellow Bark.

From a variety of experiments performed by Mr. Battley, the following conclusions are drawn.

1. Bark will yield to *cold distilled water* all its constituents except starch and woody fibre, some earthy salts, and a small portion of tannin and quinine, which can only be separated from the tissue by means of an acid.

2. 28 lbs. of good yellow bark will yield from 5 to 6 lbs. of concentrated liquor, sp. gr. 1200, containing about 10 oz. quinine; the aroma and the greater part of the tannin and iron, and the peculiar acid of bark, of which only a small portion is lost, forming an inert salt with lime.

3. To form this liquor it is only necessary to sub-pulverize the bark, and macerate it from four to six hours in twice its weight of cold distilled water, repeating the process twice or at most thrice; to concentrate the infusions over a water-butt to sp. gr. 1200, and allow the liquor to deposit the gummy matter and so much of the tannin as it cannot retain in solution.

To separate the gum that may still remain in the liquor, and to prevent any decomposition, proof spirit is added to it, until the sp. gr. of the liquor is reduced to 1100. The quinine still remaining in the bark, may, if it be thought desirable, be separated by acetic acid, and precipitated from its solution by ammonia; and being re-dissolved in a small quantity of dilute acetic acid, may be diffused in the liquor.

The advantages of this medicine are—

1. That it contains not one but all the active principles of yellow bark.

2. That the greater part of the quinine is preserved *in its natural state* in combination with the peculiar acid of bark, in which it is more soluble than in sulphuric acid.

3. That the active principles *have undergone no change,* either in the exposure to too great heat, as in the decoction, &c. or by being brought into too close contiguity, as in the extract, in which secondary formations take place to so great an extent that water is incapable of re-dissolving it.

4. That, containing no starch and little gum, it will remain unaltered for a great length of time.

5. That the quantity of spirit contained in a dose is too small to be objectionable.

6. That it is a convenient, agreeable, and elegant medicine, miscible with wine or water in any proportions.

The above is a *pharmaceutical* analysis of yellow bark; Mr. Battley hopes soon to present a *chemical* analysis, as well as a similar set of experiments on the cinchona lancifolia.—(*Medical Gazette,* April 28th.)

---

### THERAPEUTIC PROPERTIES OF MATICO. By DR. HUNTER LANE.

The leaves of a tree called *matico,* growing wild in Peru, described by Dr. Martius, in the Pharm. Central. Blatt. Jan. 1843, as a "Phlomis," but delineated in the "Flora Peruviana," as a *piper angustifolium,* have long enjoyed celebrity among the Indians for their styptic properties, when applied to bleeding surfaces, as also for their aphrodisiac virtues, when administered internally. Their styptic powers, as an external application, have been fully established here. The leaves, the parts employed, are acuminated, lanceolate, slightly crenate, deeply wrinkled, of a dark green colour on the upper, and a pale green on the lower surface. They vary from three to six inches long, and from half an inch to an inch or more wide. From these leaves Dr. H. Lane had two preparations made; an infusion thus:—

℞. Matticonis Foliorum unciam; Aquæ destillatæ ferventis octarium. Macera per horas duas in vase leviter clauso et cola.

A Tincture thus:—

℞. Matticonis Foliorum uncias duas cum semisse; Spiritûs Tenuioris octarium. Macera per horas quatuordecim et cola.

These preparations have a pale green colour, faint aromatic odour, with a slightly astringent taste.

Dr. Lane has prescribed this remedy in some forms of chronic diarrhœa, but with uncertain results. In leucorrhœa he thought it realized all the objects derivable from the topical employment of the nitrate of silver injections, but with three superior advantages over that agent. It is more expeditious in its action, less variable in its operation, and more cleanly in its use. In the acute form of the disease it should not be ever employed. But in the chronic form, depending on diminished tone and necessary congestion of the secernent capillaries of the mucous lining of the respective parts of the sexual apparatus, whence the discharge proceeds, stimulating and astringent injections are generally found useful and effective. It is for cases of this kind of leucorrhœa, whether uterine or vaginal, that the Doctor recommends the matico as an injection. He has also employed it successfully in that description of varicose and ulcerated condition of the rectum for which Dr. Houston has proposed the topical application of nitric acid. Among the many diseases in which it would seem that this remedy is worthy of trial may be enumerated hæmatemesis, hæmoptysis, dysentery, and certain forms of hæmaturia.—(*Medical Gazette,* Oct. 6, 1843.)

---

### ON THE SOLUBILITY AND SOME OTHER PROPERTIES OF SULPHATE OF POTASH. By MR. T. REDWOOD.

Chemists differ very much with regard to the extent of solubility of sulphate of potash in water. To settle the question, Mr. Redwood instituted some experiments, partly with sulphate of potash alone, some with the addition of a quantity of sesquicarbonate of soda, equal to half the quantity of sulphate of potash employed; and some, with the addition of bicarbonate of potash, equal to one-half the sulphate of potash. The inferences from these experiments were—that one part of sulphate of potash requires 11.63 parts of water for its solution at 63°; whereas one part of sulphate of potash, mixed with half its weight of sesquicarbonate of soda, is soluble in 8.74 parts of water at the same temperature. It appears also that whilst the presence of sesquicarbonate of soda increases the solubility of sulphate of potash, a contrary effect is produced by the presence of bicarbonate of potash: the same amount of water being necessary in the latter case, as the two salts would require for their solution separately. The state of aggregation of sulphate of potash appears to influence its solution very considerably. The increased solubility of sulphate of potash, when mixed with sesquicarbonate of soda, arises from the partial decomposition of these two salts, according to Mr. Redwood. This is strictly in accordance with what happens in many analogous cases; thus, sulphate of barytes may be decomposed by carbonate of potash. These experiments by Mr. Redwood were undertaken in consequence of the recent case of poisoning with sulphate of potash. The poisonous influence of sulphate of potash has been ascribed to its insolubility; this is somewhat strange, as insolubility is, in many instances, a preventive of poisonous effects. Dr. A. T. Thompson, at the Pharmaceutical Society, where the matter was discussed, set the matter in a clear light by defining a poison to be a "substance, which, administered in small quantity, produces deleterious or fatal results." So that sulphate of potash can never be numbered in the list of poisons, in as much as "almost any substance would act injuriously, if taken in a poisonous dose." Did such a statement come from a less eminent authority, it would have a chance of being set down as what Mr. Locke calls an "identical proposition."—(*Pharmaceutical Journal*, Dec. 1843.)

---

### Ceratum Saponis. By Dr. Houlton.

Dr. Houlton observed that this preparation, being generally employed as an external dressing, requires to be of a much firmer consistence than a common cerate, in order to enable it to keep the under-dressings *in situ*; yet much of that sold in the shops is soft and very deficient in adhesiveness. The chairman (of the Pharm. Society) pointed out that there were two specimens of this preparation kept, one *hard*, and the other *soft*, the former passing under the name of Emplastrum Cerati Saponis. He had received two formulæ from members of the Society, which were as follows:—

### CERATUM SAPONIS DURUM.

Liq. Plumb. Acet. ℔18.

Evaporate to ℔7.—then add—

Sap. Castil. ℔2.

Ol. Oliv. ℔4.

Ceræ Flavæ, ℔5.

### HARD SOAP CERATE.

Take Black Vinegar, 1 gallon.
(The residuum of common vinegar after distillation.)
Yellow Wax, 2 pounds 8 ounces.
Olive Oil, 4 pounds.
Castile Soap, 2 pounds.
Litharge, in powder, 4 pounds.

Dissolve the soap in the vinegar; then add the litharge by degrees, and keep it stirring till the vinegar is absorbed. The wax must be dissolved in the oil, then added to other articles, and boiled to a proper consistence.

*Pharmaceutical Journal*, Dec. 1843.

### PROVINCIAL SCHOOL OF PHARMACY.

The Pharmaceutical Society are in earnest, and are taking the way to exercise, at some future time, great influence. Their present object is to establish country Schools of Pharmacy. The Council have passed the following Resolutions :—

"1st. That Country Schools be assisted in such towns or districts as can show to the Council a prospect of effecting this object, according to some satisfactory plan, and that the grants awarded in each case be equal to one fourth of the amount of annual subscriptions received from the town or district.

"2d. That in the establishing or assisting of Country Schools, such schools shall be considered as emanating from the parent Society, and shall be designated BRANCH SCHOOLS OF THE PHARMACEUTICAL SOCIETY OF GREAT BRITAIN."

An Annual grant of money—central control on the part of the Council—Lectures on Chemistry, Materia Medica, Medical Botany, and Pharmacy—a Library—and a Collection of Materia Medica are the principal features of the plan.—(*Pharmaceutical Journ.* Dec. 1, 1843.)

---

### THE PROFITS OF PHARMACEUTICAL CHEMISTS.

The last Number of the Pharmaceutical Journal contains a clever, and what is more, a sensible article on this subject. Without going into the reasoning, we may content ourselves with the conclusions at which the writer arrives, which are two :—

First, that the Pharmaceutical Chemists of this country are very inadequately remunerated for their labour.

Secondly, that the *actual cost* of the Druggist's stock is resolved into several parts, of which the price originally paid for the drugs in their simple state, forms so small a proportion, that the actual difference between the expense of medicine of the best and second quality, is very much less than the apparent difference according to the prime cost of the article. Consequently, the saving which is effected by the purchase of inferior drugs is much more insignificant than is generally supposed, and is a very paltry return for the loss of character resulting from such practices.—(*Ibid.*)

---

### HYDRATED PEROXIDE OF IRON IN POISONING BY ARSENIC.

To a young woman, supposed to have swallowed about five drachms of white arsenic, one grain and a half of tartar emetic was given, and a clyster with three ounces of olive oil. After one minute, vomiting took place, of a greenish liquid and a little blood, in which arsenious acid was detected. Immediately the hydrated peroxide of iron, in a large dose, was commenced, and continued for several hours, till 2½ pounds of it had been taken. After each dose renewed vomitings occurred, by which all the poison seems to have been dislodged. In the evening the use of an emollient ptisan, containing some nitre, was followed by a copious stool and an abundant diuresis. The hydrated peroxide was continued, and in eight days the patient was convalescent.—*Gaz. des Hôpitaux,* Aug. 15.

